# Development and validation of a patient-reported questionnaire assessing systemic therapy induced diarrhea in oncology patients

**DOI:** 10.1186/s12955-017-0794-6

**Published:** 2017-12-22

**Authors:** Michelle Lui, Daniela Gallo-Hershberg, Carlo DeAngelis

**Affiliations:** 1 Hamilton Health Sciences Centre, Hamilton, ON Canada; 2 Leslie Dan Faculty of Pharmacy, University of Toronto, Toronto, ON Canada; 3 North York General Hospital, Toronto, ON Canada; 4 Sunnybrook Odette Cancer Centre, Toronto, ON Canada

**Keywords:** Diarrhea, Assessment, Patient-reported, Questionnaire, Validation, Supportive care, Quality of life

## Abstract

**Background:**

Systemic therapy-induced diarrhea (STID) is a common side effect experienced by more than half of cancer patients. Despite STID-associated complications and poorer quality of life (QoL), no validated assessment tools exist to accurately assess STID occurrence and severity to guide clinical management. Therefore, we developed and validated a patient-reported questionnaire (STIDAT).

**Methods:**

The STIDAT was developed using the FDA iterative process for patient-reported outcomes. A literature search uncovered potential items and questions for questionnaire construction used by oncology clinicians to develop questions for the preliminary instrument. The instrument was evaluated on its face validity and content validity by patient interviews. Repetitive, similar and different themes uncovered from patient interviews were implemented to revise the instrument to the version used for validation. Patients starting high-risk STID treatments were monitored using the STIDAT, bowel diaries and EORTC QLQ-C30. The STIDAT was evaluated for construct validity using exploratory factor analysis (EFA) using minimal residual method with Promax rotation, reliability and consistency. A weighted scoring system was developed and a receiver-operating characteristic (ROC) curve evaluated the tool’s ability to detect STID occurrence. Median scores and variability were analysed to determine how well it differentiates between diarrhea severities. A post-hoc analysis determined how diarrhea severity impacted QoL of cancer patients.

**Results:**

Patients defined diarrhea based on presence of watery stool. The STIDAT assessed patient’s perception of having diarrhea, daily number of bowel movements, daily number of diarrhea episodes, antidiarrheal medication use, the presence of urgency, abdominal pain, abdominal spasms or fecal incontinence, patient’s perception of diarrhea severity, and QoL. These dimensions were sorted into four clusters using EFA – patient’s perception of diarrhea, frequency of diarrhea, fecal incontinence and abdominal symptoms. Cronbach’s alpha was 0.78; kappa ranged from 0.934–0.952, except for abdominal spasms (κ = 0.0455). The positive predictive value was 96.4%, with the minimum score of 1.35 predicting a positive STID occurrence. Patients with moderate or severe diarrhea experience significant decreases in QoL compared to those with no diarrhea.

**Conclusions:**

This is the first patient-reported questionnaire that accurately predicts the occurrence and severity of diarrhea in oncology patients via assessing several bowel habit dimensions.

## Background

Systemic therapy-induced diarrhea (STID) is a common side effect experienced by cancer patients. While the incidence varies among regimens, it is estimated that up to 50% to 96% of patients treated with high-risk chemotherapies or targeted agents (agents in which diarrhea is among the most common side effects experienced by patients) experience STID, with up to 5–30% of those patients suffering from Grade 3 or higher diarrhea. [1–4] In many patients, STID can worsen patients’ quality of life by causing emotional distress, decreased level of social functioning, hindering work attendance and prevent travel. [1, 5] Patients with severe STID are at particularly high risk of dehydration and electrolyte disturbances [1, 6, 7], which frequently lead to treatment delays or dose reductions that may make treatments less efficacious. [5, 8] Diarrhea-associated mortality has been reported to be up to 3.5% in studies in which colorectal cancer patients treated with irinotecan and 5-fluorouracil. [9, 10] Therefore, prompt and accurate assessment of STID is warranted to prevent complications and deterioration of quality of life, and optimize patient outcomes.

Currently, the National Cancer Institute - Common Terminology Criteria for Adverse Events (NCI-CTCAE) is the accepted scale in evaluating severity of STID in clinical trials. It evaluates severity based on increases in bowel movement frequency or ostomy output, the presence of incontinence or the inability to perform self-care activities of daily living. [5, 8, 11] However, there are multiple limitations restricting its use in clinical practice. It does not consider the presence of other diarrhea-related signs and symptoms, including stool form, duration of diarrhea, abdominal pain, urgency or a decrease in quality of life scores. The CTCAE was also designed as a clinician-reported questionnaire, which have two main drawbacks. Clinician-reported questionnaires are thought to overestimate or underestimate diarrhea severity in 73% of cancer patients compared to patient-reported diarrhea severity. [12] Moreover, they elicit less information about toxicities compared to patient-reported questionnaires. [13–15] Furthermore, the CTCAE has never been formally validated despite being widely used for more than thirty years.

The absence of other assessment tools to address these issues prompted institutions to create their own algorithms in attempts to standardize a comprehensive approach to assess STID. While they identify other criteria in addition to those in the CTCAE to guide clinicians in their assessments, it is not known whether they give rise to valid evaluations of diarrhea severity, as these algorithms also have not been validated. In addition, these algorithms were not designed to perform the assessment themselves, but were intended to act as prompts to assist clinicians in their practice. This also leads to considerable practice variation in identifying the occurrence and evaluating the severity of STID, which contributes further towards the assessment inaccuracies of STID severity.

Evidently, there is a need for a standardized tool that can accurately and reliably determine the presence and severity of STID. [8] Besides stool frequency or presence of incontinence, bowel habits should also be evaluated based on assessment of stool consistency, urgency, abdominal pain, duration of diarrhea and accompanying symptoms, impact on self-care, use and effect of medications and assessment of impact of STID on quality of life. [8] The tool should be patient-reported to overcome challenges of accuracy in evaluating severity of STID in clinician-reported tools. [12, 14–16] Finally, the tool must be constructed to meet the needs of patients and subsequently validated in cancer patients using a wide variety of systemic therapies to ensure robustness and validity in the information gathered from the tool. To address the need for a new STID assessment tool, we developed a patient-reported diarrhea assessment tool to be used in cancer patients actively treated with chemotherapy or systemic therapies that are considered high-risk to cause diarrhea. We report the development and internal validation of the Systemic Therapy Induced Diarrhea Assessment Tool (STIDAT).

## Methods

This prospective study was designed in two phases using the Iterative Process used to develop a patient-reported outcome (PRO) instrument. [17] The development phase aimed to create the STIDAT and to gain the patient perspective in their assessment of STID. It consisted of hypothesizing the conceptual framework, drafting the instrument and confirming the framework. Findings from the development phase were used to complete the validation phase in which the STIDAT was internally validated in cancer patients actively treated on systemic therapy. The study was performed in accordance with the ethical standards of the Research Ethics Board committees of Sunnybrook Health Sciences Centre and North York General Hospital, as well as with the 1964 Helsinki declaration and its later amendments or comparable ethical standards. Informed consent was obtained from all individual participants included in the study, which includes consent to participate in the study, and to publish the data collected from participants.

### Development phase (phase I): Study design and participants

In the first step of hypothesizing the conceptual framework, the population of interest and intended application of use were conceptualized by the investigators. Item generation was completed from a literature review, interviews with clinicians and patient interviews. A literature search was conducted to determine published diarrhea assessment tools used in any patient population, and to identify potential bowel assessment dimensions that can be used to construct the assessment tool. The search was conducted in PubMed and EMBASE using [diarrhea AND assess* AND question*] OR [diarrhea AND clinical assessment tool*] as MeSH words. References of selected papers were scanned for additional tools that may not have surfaced from the search. The literature search was also extended to include any unpublished tools that other institutions or associations may have developed and made available for clinical use. The search was limited to studies in the English language. The tools were initially selected by the primary author (ML) and subsequently reviewed by the last author (CDA). Studies that provided only general guidelines, criteria or algorithm of diarrhea assessment rather than the questions from a tool were excluded.

An expert panel of oncology healthcare practitioners reviewed the findings from the literature review to select bowel assessment dimensions important for bowel habit assessment, develop questions for each dimension that clinicians collectively perceived as important for bowel habits assessment and agree upon appropriate wording and format of questions. Furthermore, the panel reviewed how well the questions individually and together evaluated the presence and severity of diarrhea. This process was used to develop the preliminary framework, as well as its face validity and construct validity from the clinicians’ perspective.

To adjust the preliminary framework, the investigators conducted patient interviews to evaluate face and content validity of the preliminary instrument from the patient perspective. Actively treated patients over eighteen years of age with a cancer diagnosis who had a history of STID within the last seven days were eligible to be interviewed. Patients experiencing diarrhea before starting treatment, had a gastrointestinal stoma or had cognitive impairment were excluded. A series of questions were asked regarding each item by the primary author to elicit information about patients’ understanding of the questions’ meaning, clarity of questions, criteria perceived to be important or unimportant for the assessment of STID and other questions that should be added or excluded from the questionnaire. Face validity was evaluated by questions like, “Would these questions be important for us to better understand your diarrhea?” while content validity was evaluated by questions like, “What do you think this question is trying to ask you?” Patients were instructed to answer the questions based on the preliminary instrument, which was reviewed during the interview.

Answers were transcribed and compared by two investigators to identify themes that would be used for revisions of the draft STIDAT. The authors performed thematic analyses by finding repetitions, similarities and differences within the answers of the patient interviews. [18] More patients were recruited if additional ideas were elicited from the most recent interview. Once saturation has been reached, no more patients were recruited and all revisions were made to the preliminary instrument. The final version was subsequently used for internal validation in the validation phase.

### Validation phase (phase II)

#### Study design and participants

The validation phase was designed to confirm the preliminary instrument designed in the development phase and to assess its reliability and validity. Adult patients starting on a regimen that included at least one high-risk chemotherapy (irinotecan, 5-fluorouracil, capecitabine) or targeted agent (erlotinib, gefitinib, afatinib). Patients who had diarrhea before the start of treatment were included. Patients were excluded if they had a gastrointestinal stoma, had cognitive impairment, had limited language proficiency in English, or had already been on a regimen that included a high-risk chemotherapy or targeted therapy. Patients completed the baseline STIDAT on the first day of treatment and the follow-up STIDAT on a weekly basis for the first cycle. The frequency of completing the STIDAT in the first cycle differed based on the treatment regimen the patient underwent. For example, patients had completed the questionnaire three times in treatment cycles that were three weeks long. Patients completed the STIDAT for the entire treatment regimen if their treatment only had one cycle. In addition, patients completed a diarrhea diary daily and the EORTC QLQ-C30 weekly. Any cases of diarrhea identified and assessed using the STIDAT were communicated and managed by the attending physician of the patients.

### Statistical analyses

Given the ratio of observations to each item is generally accepted to be at least 5 to 1 to prevent overfitting [19], the number of dimensions assessed in the STIDAT was expected to be ten based on the preliminary instrument, and a dropout rate of 40%, the number of patients who needed to be recruited was calculated to be 70 and the number of patients who needed to complete the validation phase was 50.

Upon completion of data collection, results of the STIDAT were compared with results of the diaries and EORTC QLQ-C30 for each patient. Internal consistency was evaluated by calculating Cronbach’s alpha. A questionnaire with an alpha coefficient of greater than 0.7 was interpreted to have good internal consistency. Furthermore, inter-rater diagnostic reliability was assessed by determining Fleiss’ kappa for binary parameters of bowel habit assessment, Pearson’s coefficient of correlation for continuous parameters and Kendall’s tau for ordinal parameters. A Fleiss’ kappa of at least 0.70 suggests acceptable inter-rater reliability among binary parameters, a large value of Pearson’s coefficient of correlation represents good correlation of continuous variables, and a high Kendall’s tau signifies high probability of concordant pairs of ordinal data. Concurrent validity of the STIDAT was evaluated by using Chi-Square test to compare STID occurrence during treatment reported in the STIDAT and EORTC QLQ-C30.

A weighted scoring system of the STIDAT was constructed using exploratory factor analysis (EFA) using the minimal residual method using “Promax” as the oblique rotation method. [20, 21] The number of factors were initially determined using eigenvalues greater than one method and confirmed using parallel analysis, with eigenvalues generated using principal components analysis (PCA). EFA was used to determine the total weight that each dimension had on the STIDAT’s ability to detect the occurrence of diarrhea. The score of each dimension was calculated by multiplying the weight with the value that corresponds to the result of its STIDAT question(s) and the total STIDAT score was determined by summing the scores of each dimension.

The median STIDAT scores of patients with and without diarrhea, as well as those by each level of patient-reported STID severity were determined. The Welch’s T-test was used to determine whether STIDAT scores of patients with and without diarrhea were significantly different from each other. To evaluate the predictive ability of the STIDAT, a receiver-operating characteristic (ROC) curve was constructed to determine the threshold (minimum STIDAT score) that predicts for STID occurrence with the highest possible values of sensitivity and specificity. The corresponding values of threshold score, sensitivity, specificity, positive predictive value (PPV) and negative predictive value (NPV) are also reported. All statistical analyses were performed using R (version 3.3.1).

As a post-hoc analysis to determine how patients perceive severity of diarrhea, we investigated whether there were any differences in quality of life among patients with no diarrhea, mild diarrhea, moderate diarrhea and severe diarrhea. Multiple comparisons were adjusted using the Bonferroni correction method. Furthermore, we determined whether there was a difference in diarrheal episodes among patients with different severities of diarrhea using the Dunnett-Tukey-Kramer multiple comparison test that was adjusted for unequal variances and unequal sample sizes.

## Results

### Development phase (phase I)

**Fig. 1:**
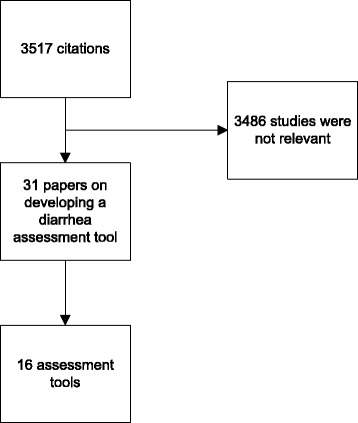
Results of the literature search

From the 16 tools uncovered from the literature review (Fig. 1), the expert panel determined the following dimensions to be relevant to assessing the presence and severity of diarrhea: patient’s perception of having diarrhea, daily number of bowel movements, daily number of diarrhea episodes, use of antidiarrheal medications, the presence of urgency, abdominal pain, abdominal spasms, fecal incontinence, patient’s perception of diarrhea severity, and quality of life. Quality of life was evaluated based on the impact of having diarrhea on the patient’s social life, mood, family life, ability to perform daily activities of living and energy level. In addition, the expert panel had included a checklist of foods and beverages that were thought to influence bowel habits by either helping or exacerbating ongoing diarrhea, with the thought that the presence of such items in patients’ diets may influence the severity of their diarrhea. A list of additional symptoms indicative of severe diarrhea, including the presence of nausea, fever and melena, was included in the questionnaire, as the members perceived these symptoms to be important in assessing severity of diarrhea.

Five patients were interviewed; saturation was achieved from their collective data. Patients generally understood why the questions were asked and correctly interpreted that they were to assess different “aspects” of their diarrhea, therefore confirming the tool’s face validity. With respect to the clarity of questions, all patients perceived the wording to be clear and provided interpretations akin to the intent of each question. Patients agreed unanimously that the questionnaire had included all the dimensions required to evaluate the incidence and severity of STID and was thus perceived to be comprehensive in STID assessment. All patients answered that each question was important to assess for the presence and severity of diarrhea, and that they would not eliminate any question from the questionnaire. Furthermore, none of the patients had additional questions to the questionnaire.

In addition, 80% of patients agreed that the definition of diarrhea was mostly driven by the presence of watery stool rather than by stool frequency. Patient 1, who reported having 5 to 6 bowel movements daily, commented that he “would have diarrhea every day if the definition was based on number of stools”, but understands that “they [were] still normal bowel movements given that [he] has normal stool each time”. Furthermore, 2 patients commented that evaluation of stool form should include forms associated with constipation in addition to diarrhea.

**Fig. 2:**
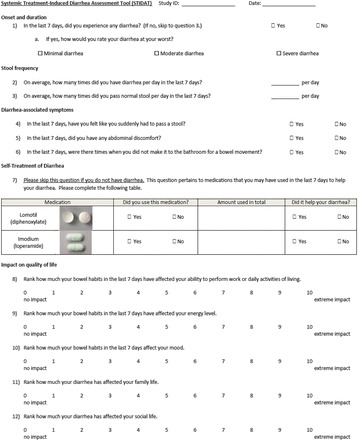
The Systemic Treatment-Induced Diarrhea Assessment Tool (STIDAT)

While the panel members initially perceived a list of foods and beverages that help or exacerbate diarrhea included in the questionnaire to be helpful to patients to evaluate their bowel habits, most patients found it difficult determining how their diet could have influenced their bowel habits. Furthermore, patients also found the checklist of symptoms of severe diarrhea to be confusing with other treatment-related toxicities. Therefore, both sections were eliminated from the preliminary instrument. The final instrument is illustrated in Fig. 2.

### Validation phase (phase II)

Eighty–nine patients were enrolled and 52 patients completed the STIDAT (Table 1). 12 withdrew consent, 20 did not complete the questionnaire and 5 were lost to follow-up.

**Table 1:** Baseline characteristics

Demographics		N (%)
Age		Median = 58 (30–88)
Male, female		26/70 (37%), 44/70 (63%)
Median treatment cycles (min-max)		1 (1–5)
Therapy		
	Chemoradiation	16/69 (23%)
	Chemotherapy alone	53/69 (77%)
Chemotherapy		
	5-fluorouracil	42/70 (60%)
	5-fluorouracil and irinotecan	8/70 (11%)
	Capecitabine	12/70 (17%)
	Erlotinib	4/70 (6%)
	Gefitinib	3/70 (4%)
	Afatinib	1/70 (1%)
Ethnicity		
	North American	26/70 (37%)
	European	22/70 (31%)
	African	4/70 (6%)
	Asian	18/70 (26%)
Cancer site		
	Colorectal	26/72 (38%)
	Breast	17/72 (24%)
	Lung	8/72 (11%)
	Pancreatic	6/72 (8%)
	Anal	5/72 (7%)
	Gastric	4/72 (6%)
	Appendix	1/72 (1%)
	Endometrial	1/72 (1%)
	Esophageal	1/72 (1%)
	Melanoma	1/72 (1%)
	Vulva	1/72 (1%)
Educational level		
	Post-secondary	47/69 (68%)
	Secondary	16/69 (23%)
	Doctorate	3/69 (4%)
	Primary	2/69 (3%)
	Post-graduate	1/69 (2%)

### Construct validity of questionnaire

Table 2 summarizes the reliability and consistency of the STIDAT. The questionnaire has an overall raw Cronbach’s alpha of 0.78 (95% CI = 0.74–0.83). Regarding the reliability of STIDAT, high kappa values were calculated between the questionnaire and diaries for the patient’s perception of diarrhea (κ = 0.934) and a positive history of antidiarrheal medication use (κ = 0.952) and a low kappa value was calculated for the dimension of abdominal spasms (κ = 0.0455). Good correlation between the questionnaire and diaries was noted for the dimensions of diarrhea severity and quality of life. Furthermore, STID occurrence reported in the STIDAT and EORTC QLQ-C30 are significantly related to each other (χ^2^ = 79.71, *p* < 0.05).

**Table 2:** Interrater reliability and consistency of STIDAT

Fleiss’ kappa			
Variable	N	Kappa	*p*-value
Diarrhea	91	0.934	*p* = 0
Urgency	113	0.623	*P* < 0.05
Spasms	113	0.0455	*P* = 0.63
Abdominal pain	113	0.571	*P* < 0.05
Fecal incontinence	113	0.685	*P* < 0.05
Medications for diarrhea	113	0.952	*P* = 0
Cronbach’s alpha			
Raw Cronbach’s alpha	0.78 (0.74–0.83)		
Standard Cronbach’s alpha	0.85		
Pearson’s Coefficient of Correlation (r^2^)			
Number of diarrhea episodes	0.377		
Total number of bowel movements	0.262		
Quality of life	0.792		

### Analysis of diarrhea symptoms and quality of life

A breakdown of signs and symptoms based on the severity of diarrhea (no to severe diarrhea) is available in Table 3. Of note, the mean overall quality of life scores (calculated as the average of the five quality of life dimensions measured in the STIDAT) were 8.95 (95% CI = 8.50–9.40) in the no diarrhea group, 8.12 (95% CI = 7.38–8.87, *p* = 0.6164) in the mild diarrhea group, 7.1 (95% CI = 6.18–8.02) in the moderate diarrhea group and 4.44 (95% CI = 1.53–7.35) in the severe diarrhea group.

**Table 3:** Signs and symptoms of diarrhea by STIDAT dimension

Dimension			All patients	No diarrhea	All patients with diarrhea	Mild diarrhea	Moderate diarrhea	Severe diarrhea
Patient’s perception of having diarrhea								
	STIDAT							
		Diarrhea	54/113 (47.8%)	0/56 (0%)	54/57 (94.7%)	18/21 (85.7%)	30/30 (100%)	6/6 (100%)
		No diarrhea	59/113 (52.2%)	56/56 (100%)	3/57 (5.3%)	3/21 (14.3%)	0/30 (0%)	0/6 (0%)
	Diary							
		Diarrhea	47/113 (41.6%)	0/56 (0%)	47/57 (82.5%)	16/21 (76.2%)	26/30 (86.7%)	5/6 (83.3%)
		No diarrhea	44/113 (38.9%)	39/56 (69.6%)	5/57 (8.8%)	3/21 (14.3%)	2/30 (6.7%)	0/6 (0%)
		Unevaluable	22/113 (19.5%)	17/56 (30.4%)	5/57 (8.78%)	2/21 (9.5%)	2/30 (6.6%)	1/6 (16.7%)
Mean number of bowel movements (SD)								
	STIDAT		1.90 (1.31)	1.55 (0.91)	2.23 (1.55)	1.60 (0.89)	2.18 (1.43)	4.58 (1.80)
	Diary		1.90 (1.34)	1.78 (1.18)	1.99 (1.50)	1.72 (1.42)	1.89 (1.30)	3.37 (2.13)
Mean number of diarrheal episodes (SD)								
	STIDAT		0.92 (1.46)	0.003 (0.019)	1.90 (1.60)	1.11 (0.82)	1.83 (1.22)	4.58 (2.20)
	Diary		0.73 (1.30)	0.11 (0.44)	1.38 (1.56)	0.99 (1)	1.22 (1.23)	3.41 (3.40)
Presence of urgency								
	STIDAT							
		Present	58/113 (51.3%)	14/56 (25%)	44/57 (77.2%)	16/21 (76.2%)	23/30 (76.7%)	5/6 (83.3%)
		Absent	55/113(48.7%)	42/56 (75%)	13/57 (22.8%)	5/21 (23.8%)	7/30 (23.3%)	1/6 (16.7%)
	Diary							
		Present	64/113 (57.1%)	17/56 (30.4%)	46/57 (80.7%)	14/21 (66.7%)	26/30 (86.7%)	6/6 (100%)
		Absent	48/113 (42.9%)	39/56 (69.6%)	9/57 (15.8%)	5/21 (23.8%)	4/30 (13.3%)	0/6 (100%)
		Unevaluable	1/113 (0%)	0/56 (0%)	2/57 (3.6%)	2/21 (9.5%)	0/30 (0%)	0/6 (100%)
Presence of abdominal discomfort								
	STIDAT							
		Present	52/113 (46.0%)	18/56 (32.1%)	34/57 (59.6%)	9/21 (42.9%)	19/30 (63.3%)	6/6 (100%)
		Absent	61/113 (55.0%)	38/56 (67.9%)	23/57 (40.4%)	12/21 (57.1%)	11/30 (36.7%)	0/6 (100%)
	Diary							
		Present	63/113 (55.8%)	22/56 (39.3%)	41/57 (71.9%)	11/21 (52.4%)	24/30 (80%)	6/6 (100%)
		Absent	49/113 (43.4%)	34/56 (60.7%)	15/57 (26.3%)	9/21 (42.9%)	6/30 (20%)	0/6 (0%)
		Unevaluable	1/113 (0.8%)	0/56 (0%)	1/57 (1.8%)	1/21 (4.7%)	0/30 (0%)	0/6 (0%)
Presence of abdominal spasms								
	STIDAT							
		Present	39/113 (34.2%)	11/56 (19.6%)	28/57 (49.1%)	9/21 (42.9%)	14/30 (46.7%)	5/6 (83.3%)
		Absent	75/113 (66.8%)	45/56 (80.4%)	29/57 (50.9%)	12 (57.1%)	16/30 (53.3%)	1/6 (16.7%)
	Diary							
		Present	10/113 (8.8%)	4/56 (7.1%)	6/57 (10.5%)	2/21 (9.5%)	3/30 (10%)	1/6 (16.7%)
		Absent	102/113 (90.3%)	52/56 (92.9%)	50/57 (87.7%)	18/21 (85.7%)	27/30 (90%)	5/6 (83.3%)
		Unevaluable	1/113 (0.9%)	0/56 (0%)	1/57 (1.8%)	1/21 (4.8%)	0/30 (0%)	0/6 (0%)
Presence of fecal incontinence								
	STIDAT							
		Present	13/113 (11.5%)	4/56 (7.1%)	9/57 (15.8%)	2/21 (9.5%)	6/30 (20%)	1/6 (16.7%)
		Absent	100/113 (88.5%)	52/56 (93.9%)	48/57 (84.2%)	19/21 (90.5%)	24/30 (80%)	5/6 (83.3%)
	Diary							
		Present	12/113 (10.6%)	2/56 (3.6%)	10/57 (17.5%)	2/21 (9.5%)	7/30 (23.3%)	1/6 (16.7%)
		Absent	100/113 (88.5%)	54/56 (96.4%)	45/57 (79.0%)	17/21 (81.0%)	23/30 (76.7%)	5/6 (83.3%)
		Unevaluable	1/113 (0.9%)	0/56 (0%)	2/57 (3.5%)	2/21 (9.5%)	0/30 (0%)	0/6 (0%)
Positive use of anti-diarrheals								
	STIDAT							
		Present	7/113 (6.2%)	0/56 (0%)	7/57 (12.3%)	0/21 (0%)	6/30 (20%)	1/6 (16.7%)
		Absent	106/113 (93.8%)	56/56 (100%)	50/57 (87.7%)	21/21 (100%)	24/30 (80%)	5/6 (83.3%)
	Diary							
		Present	8/113 (7.1%)	0/56 (0%)	8/57 (14%)	0/21 (0%)	7/30 (23.3%)	1/6 (16.7%)
		Absent	103/113 (91.2%)	56/56 (100%)	47 (82.5%)	19/21 (90.5%)	23/30 (76.7%)	5/6 (83.3%)
		Unevaluable	2/113 (1.7%)	0/56 (0%)	2/57 (3.5%)	2/21 (9.5%)	0/30 (0%)	0/6 (0%)
Mean quality of life score (SD)								
	STIDAT		8.1 (2.24)	8.95 (1.70)	7.34 (2.40)	8.12 (1.63)	7.1 (2.46)	4.44 (2.77)
	EORTC QLQ-C30		6.7 (2.45)	7.25 (2.40)	6.12 (2.39)	6.57 (2.44)	6.20 (2.25)	4.36 (2.50)

Using the Dunnett-Tukey-Kramer test, it was determined that there were significant decreases in quality of life experienced by patients with moderate diarrhea (−1.86, 95% CI = −3.05, −0.67, *p* < 0.001) and severe diarrhea (−3.64, 95% CI = −5.91, −1.38, p < 0.001) compared to patients with no diarrhea. There was a trend towards a decrease in quality of life in patients with severe diarrhea compared to those with mild diarrhea (−2.82, 95% CI = −5.3, −0.37, *p* = 0.017). There was no difference in quality of life between mild and no diarrhea, mild and moderate diarrhea, and moderate and severe diarrhea patients (Table 4).

**Table 4:** Differences in STIDAT quality of life scores based on severity of diarrhea

Comparison	Difference in quality of life score	95% confidence interval	*p*-value
Mild to no diarrhea	−0.83	−2.18, 0.52	*p* = 0.38
Moderate to no diarrhea	−1.86	−3.05, −0.67	*p* < 0.001
Severe to no diarrhea	−3.64	−5.91, −1.38	*p* < 0.001
Moderate to mild diarrhea	−1.04	−2.54, 0.46	*p* = 0.27
Severe to mild diarrhea	−2.82	−5.26, −0.37	*p* = 0.17
Severe to moderate diarrhea	−1.78	−4.13, 0.58	*p* = 0.21

### Factor analysis

**Fig. 3:**
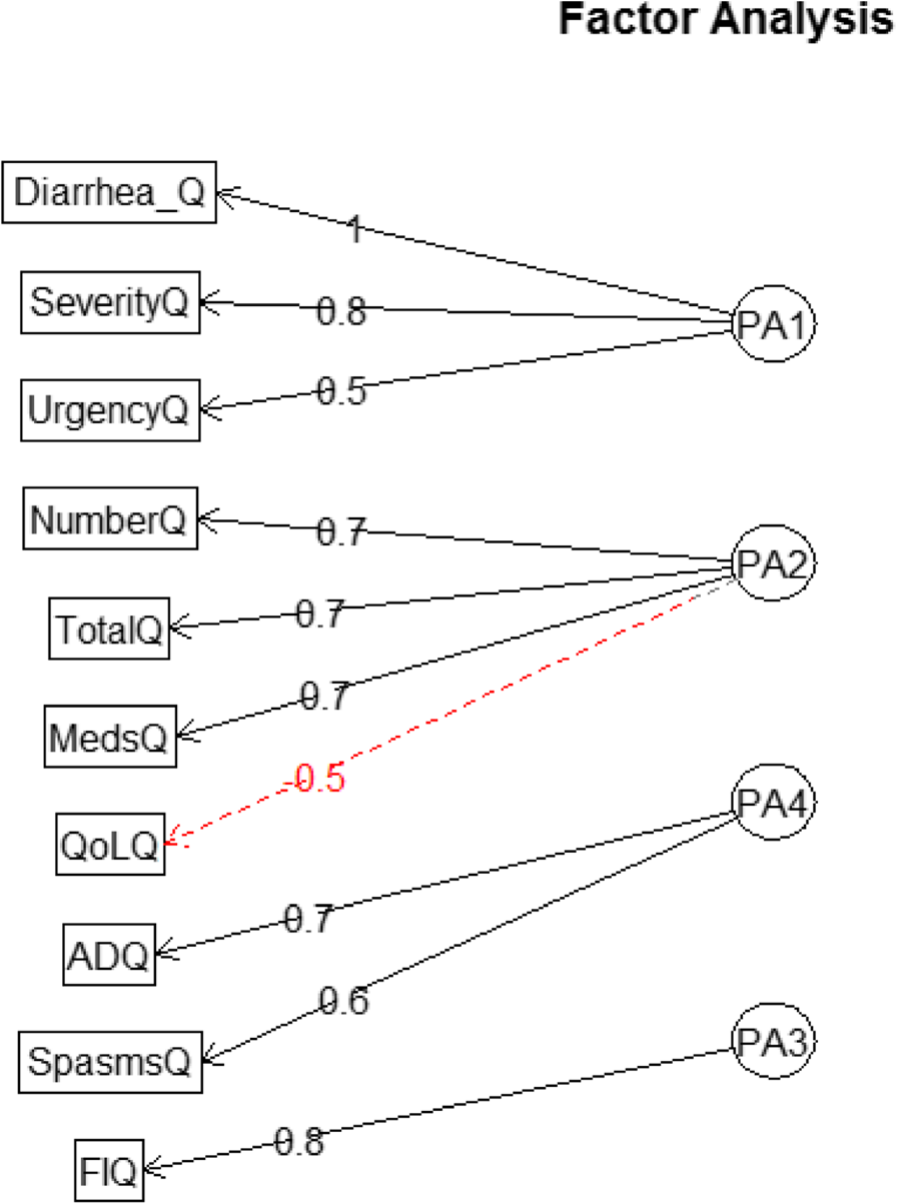
Factor analysis diagram depicting the relationships of the dimensions of the Systemic Treatment Induced Diarrhea Assessment Tool (STIDAT). Diarrhea_Q = patient’s report of diarrhea occurrence, SeverityQ = patient’s report of diarrhea severity, UrgencyQ = presence of urgency, NumberQ = number of diarrhea episodes, TotalQ = total number of bowel movements, MedsQ = use of antidiarrheal medications, QoLQ = quality of life, ADQ = presence of abdominal pain, SpasmsQ = presence of abdominal spasms, FIQ = presence of fecal incontinence. Cluster 1 (PA1) = patient perception of diarrhea; Cluster 2 (PA2) = bowel frequency, Cluster 3 (PA3) = fecal incontinence, Cluster 4 (PA4) = abdominal symptoms

Using the eigenvalues greater than one rule, the number of factors was determined to be 4. This was confirmed with the parallel analysis approach. Using the minimal residual method with Promax rotation, the factor loadings of each dimension to each factor were generated. The dimensions were grouped into four clusters. The dimensions of patient’s report of the occurrence and severity of diarrhea, and the presence of urgency were related conceptually by the patient’s perception of having diarrhea, or Cluster 1. Cluster 2 – frequency of diarrhea – comprised of total number of bowel movements, number of diarrhea episodes, quality of life and medication use. Fecal incontinence is the sole dimension of Cluster 3. Finally, abdominal spasms and abdominal pain were grouped together as abdominal symptoms – Cluster 4. Figure 3 and Table 5 shows the relationships of the dimensions assessed by the STIDAT.

**Table 5:** Factor loadings and communalities for the Systemic Treatment Induced Diarrhea Assessment Test (STIDAT)

	PPD	BHF	FI	AbS	H^2^	U^2^
Presence of diarrhea	0.98	−0.01	−0.01	0.01	0.95	0.053
Severity of diarrhea	0.77	0.39	−0.01	−0.13	0.92	0.077
Number of diarrhea episodes	0.32	0.71	−0.10	0.07	0.80	0.197
Total bowel movement episodes	−0.16	0.67	−0.09	0.39	0.62	0.382
Presence of urgency	0.37	−0.13	0.16	0.38	0.43	0.566
Presence of abdominal spasms	−0.05	0.06	0.57	0.05	0.37	0.628
Presence of abdominal pain	−0.02	−0.07	0.80	−0.10	0.52	0.478
Presence of fecal incontinence	−0.08	0.05	−0.10	0.63	0.35	0.647
Quality of life	0.02	−0.40	−0.17	−0.23	0.42	0.576
Medication use	−0.02	0.67	0.20	−0.12	0.56	0.440

PPD = patient’s perception of diarrhea, BHF = bowel habit frequency, FI = fecal incontinence, AbS = abdominal symptoms, H2 = communality, U2 = uniqueness

### Scoring system of STIDAT

Table 6 is the weighted scoring system from EFA using an eigenvalue of 4, as per the initial PCA. The multiplication of each component score with its corresponding component weight, followed by the addition of an adjustment factor, yields a total score. Patients without diarrhea had a mean score of 0.72 (SD = 0.21), while patients with diarrhea had a significantly higher score of 2.25 (SD = 0.73) (*t* = 15.04, 95% CI = 1.33–1.73).

**Table 6:** Scoring system of STIDAT

Components	Proportion explaining diarrhea	Score calculation
Factor 1: Patient’s perception of diarrhea		
Presence of diarrhea	0.193	N^1^ × 0.193
Severity of diarrhea	0.529	N^2^ × 0.529
Presence of urgency	0.048	N^1^ × 0.048
Factor 2: Bowel movement frequency		
Number of bowel movements	0.050	N^3^ × 0.050
Number of diarrhea episodes	0.161	N^3^ × 0.161
Medication use	0.060	N^1^ × 0.060
Quality of life	−0.048	Average N_1–5_ x − 0.048
Factor 3: Fecal incontinence		
Presence of fecal incontinence	0.016	N^1^ × 0.016
Factor 4: Abdominal symptoms		
Presence of abdominal spasms	0.032	N^1^ × 0.032
Presence of abdominal discomfort	0.031	N^1^ × 0.031
TOTAL		Sum of component scores +0.48^*^

N1: yes = 1, no = 0

N2: no = 0, mild = 1, moderate = 2, severe = 3

N3: an integer of *n* ≥ 0

*adjustment factor

**Fig. 4:**
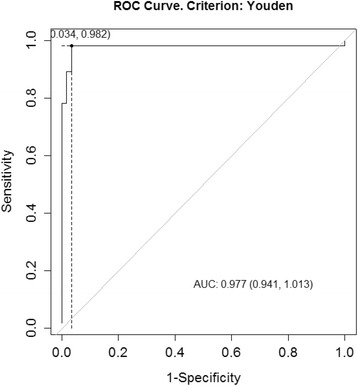
Receiver-operating characteristic (ROC) curve analysis for STIDAT’s ability to detect diarrhea occurrence

ROC analysis yielded an area-under-the-curve (AUC) of 0.977 (95% CI = 0.941–1.013) at the cut-off score of 1.35 in predicting the occurrence of diarrhea (Fig. 4). Sensitivity, specificity, PPV and NPV were 0.982, 0.966, 0.964 and 0.983, respectively.

**Fig. 5:**
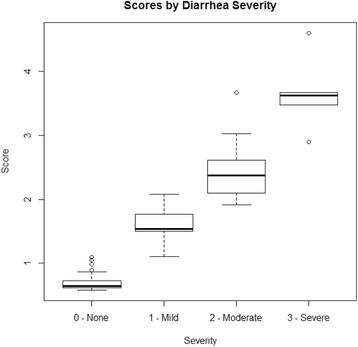
Boxplot of STIDAT stratified by diarrhea severity

Table 7 and Fig. 5 show STIDAT scores stratified by diarrhea severity. Median scores of no, mild, moderate and severe diarrhea cohorts were 0.64, 1.54, 2.38 and 3.63, respectively. Based on the ranges of scores for each severity (Table 7 and Fig. 5), clinicians may categorize scores from 0 to 1.1 as no diarrhea, greater than 1.1 to 2 as mild diarrhea, greater than 2 to 3 as moderate diarrhea and greater than 3 as severe diarrhea.

**Table 7:** STIDAT scores by diarrhea severity

	Median	Interquartile range	Minimum	Maximum
No diarrhea	0.643984	0.726803–0.618736 = 0.108067	0.57867	1.095777
Mild diarrhea	1.540987	1.774623–1.483602 = 0.291021	1.107415	2.076253
Moderate diarrhea	2.375934	2.388316–2.363551 = 0.024764	1.909746	3.674208
Severe diarrhea	3.630018	3.670862–3.475936 = 0.194926	2.899779	4.605079

## Discussion

To our knowledge, this is the first standardized patient-reported questionnaire used to assess STID in cancer patients. A wide range of cancer sites and systemic therapies were represented in this study, indicating that the STIDAT was robust enough to be used in the entire cancer patient population actively treated with any systemic therapy. Furthermore, the study also reported patients of various educational levels and ethnicities, suggesting that the STIDAT was also valid across different levels of education and ethnicities. Patients who received concurrent chemoradiation were included, as radiation has a role in intensifying the damage that chemotherapy has, including diarrhea if the radiation were directed in the lower gastrointestinal tract. Therefore, the STIDAT could also be used in patients who are treated with radiation while receiving systemic therapy.

### Study findings

The patient interviews revealed that most patients defined diarrhea based on the presence of watery stool without mention of increased stool frequency, which is a departure to how the CTCAE assesses diarrhea mostly through an increase in stool frequency. This discrepancy among the clinician and patient definitions of diarrhea may in part account for the difference in the evaluation of diarrhea severity by patients and the clinicians via the CTCAE. This finding suggests the need to choose one definition of diarrhea that patients use when reporting the occurrence and severity of STID to ensure clarity and standardization in the assessment of STID. To address this discrepancy, the STIDAT uses the patient definition in which diarrhea was identified based on the presence of watery stool.

The STIDAT was observed to have good internal consistency with a raw Cronbach’s alpha of 0.78, which far exceeded 0.7. It has similar degrees of internal consistency with other validated diarrhea assessment tools used in other disease states, including the SSC-HIV (α = 0.749), Irritable Bowel Syndrome Quality of Life (α = 0.963), DAPonDEN (α = 0.67–0.717) and Birmingham IBS symptom questionnaire (α = 0.74–0.90). [22–24] It also had good inter-rater reliability, particularly in the dimensions of patient’s report of the occurrence and severity of diarrhea, urgency, fecal incontinence and antidiarrheal medication use, suggesting that the STIDAT is particularly robust in evaluating diarrhea based on those dimensions. The STIDAT exhibited poor reliability in reporting abdominal spasms, suggesting that patients may not understand what spasms are, or are not able to differentiate between spasms and abdominal discomfort.

The weighted scoring system is based on how much each component contributes towards the incidence of diarrhea as predicted by EFA. The score of each question corresponding to its component would be multiplied by its component weight. Scores of binary variables (yes/no questions) would be either 0 (no) or 1 (yes), scores for diarrhea severity vary from 0 (no diarrhea) to 3 (severe diarrhea) and scores of questions pertaining to episode frequency would be an integer starting from 0. The quality of life score is the average of the five scores corresponding to the five dimensions of quality of life in the STIDAT. The component score for quality of life has a negative sign because the quality of life questions are ranked in reverse. The total STIDAT score is the sum of all component scores and the adjustment factor (0.48), which offsets the minimum quality of life score that a patient may reach to prevent a negative STIDAT score.

The STIDAT dimensions were grouped based on their relationships with each other. A heightened increased sense of urgency to have a bowel movement increases the perception of having diarrhea that may be more severe, hence the potential rationale for urgency, patient report of diarrhea occurrence and severity to be connected via Cluster 1. While it is apparent that total number of bowel movements and number of diarrhea episodes are related to Cluster 2, medication use can reduce bowel movement frequency and quality of life can be negatively affected from increased frequency of bowel movements. Therefore, medication use and quality of life are also grouped together into Cluster 2. While fecal incontinence is a diarrhea symptom along with abdominal spasms and pain, the former may happen independently of diarrhea occurrence. This is observed by the low component weight of fecal incontinence in the scoring system in determining the presence and severity of diarrhea in patients.

A score of 1.35 is the minimum for the STIDAT to detect whether a patient objectively has diarrhea or not with excellent sensitivity and specificity. Furthermore, the STIDAT scoring system can identify the severity level of STIDAT of patients. There was a distinct separation of scores in patients of each group of diarrhea severity. While there is some overlap between mild and moderate diarrhea, it is sufficiently small such that the potential error in differentiation is small. Because the management of mild and moderate diarrhea is similar, the potential harm on the patient is not expected to be significant. Therefore, this STIDAT can accurately identify the occurrence and severity of diarrhea through a comprehensive assessment and appropriate weighting of various bowel habit dimensions.

### Benefits and applications of STIDAT

The development of the STIDAT also highlighted the significant differences in quality of life among patients with different severities of diarrhea. Patients with severe diarrhea had significantly worse quality of life compared to patients with no or mild diarrhea. While there was no difference among patients with moderate and mild diarrhea, a significant decrease in quality of life was observed among patients with moderate and no diarrhea. Therefore, clinicians should aim to improve diarrhea management of patients with moderate to severe diarrhea with the therapeutic goal to decrease it to at least mild diarrhea, not simply to prevent dehydration and potential treatment delays, but to maintain patients’ quality of life throughout their treatment journey.

The STIDAT is superior to the CTCAE as an oncology-based clinical bowel assessment tool. As a patient-reported questionnaire, it circumvents potential underreporting or inaccuracies in clinician-based assessments in the occurrence and severity of diarrhea. This increases the likelihood of patients’ STID in being assessed and managed appropriately, which may decrease negative consequences associated with STID, including dehydration-associated hospitalizations, treatment dose reductions or delays, and decreases of quality of life associated with undermanaged STID. It assesses beyond stool frequency and presence of fecal incontinence to include frequency of diarrhea episodes, quality of life, stool form and other symptoms of diarrhea to provide comprehensive insight on the presence and severity of diarrhea. This enables clinicians to better understand the toxicity and provide more appropriate management for it. It also uses a weighted scoring system to objectively assess the presence and severity of STID using patient-reported data, which contrasts sharply from the arbitrarily set stool frequency intervals that define each CTCAE severity grade. The use of a weighted scoring system allows for objective stratification of diarrhea severity that considers multiple dimensions. In addition to providing a total score that characterizes the severity of diarrhea, it provides a score for each item to provide insight for what may drive the score, which the CTCAE cannot do with an overall grade. The STIDAT was also developed using the presence of watery stool as the definition of diarrhea – as per patient interviews – compared to the presence of increased stool frequency in the CTCAE. The use of stool form to define diarrhea seems effective in detecting diarrhea occurrence, as reflected by its excellent specificity and sensitivity. Furthermore, the STIDAT has undergone a formal development and validation process, unlike the CTCAE.

### Limitations

This study has some limitations. The validation phase was conducted in two Centres with a small, albeit varied, patient population that lacked patients with hematologic malignancies. While around 60% of recruited patients had completed the questionnaire, these patients may be particularly motivated and self-efficacious and therefore, the use of the questionnaire may be biased towards such individuals. It is not designed to be used in patients with an ostomy; thus, the STIDAT cannot assess the bowel habits of these patients. It is restricted for patients with adequate working English and cognitive ability to self-report his or her bowel habits. Finally, this assessment requires external validation to further characterize validity and predictive ability. Future validation efforts will be focused on including more centres, amassing a larger sample size, including hematology patients and modifying the questionnaire to accommodate patients with ostomies and patients speaking other languages.

## Conclusion

The STIDAT is the first validated, patient-reported assessment tool designed to accurately identify the presence of diarrhea and its severity using multiple bowel habit dimensions in patients with STID of multiple solid tumours who received systemic therapy with or without radiation. Patients with a STIDAT score of 1.35 or higher are identified to have diarrhea. Patients with a STIDAT score of 0 to 1.1 have no diarrhea, greater than 1.1 to 2 have mild diarrhea, greater than 2 to 3 have moderate diarrhea and greater than 3 have severe diarrhea. Clinicians must aim to treat cancer patients to have no or mild diarrhea to prevent a detrimental impact on quality of life.

## Abbreviations

AUC: area under the curve
CTCAE: Common Terminology Criteria for Adverse Events
EFA: Exploratory factor analysis
NCI-CTCAE: National Cancer Institute - Common Terminology Criteria for Adverse Events
NPV: Negative predictive value
PCA: Primary component analysis
PPV: Positive predictive value
ROC: Receiver-operator characteristic
STID: Systemic therapy-induced diarrhea
STIDAT: Systemic Therapy-Induced Diarrhea Assessment Test
